# What Japanese Women with Breast Cancer Decide: A Mixed Methods Analysis of Web-Based Open-Ended Responses

**DOI:** 10.31557/APJCP.2021.22.9.2909

**Published:** 2021-09

**Authors:** Keiko Yamauchi, Mitsuyo Nakashima, Motoyuki Nakao

**Affiliations:** 1 *Department of Public Health, School of Medicine, Kurume University, Fukuoka, Japan. *; 2 *Department of Nursing, School of Medicine, Fukuoka University, Fukuoka, Japan. *

**Keywords:** Breast cancer, medical care decisions, sociopsychological decisions

## Abstract

**Background:**

Living with breast cancer (BC) involves making many decisions, which immediately follow the diagnosis of BC. These decisions concern not only medical care, but also sociopsychological aspects, suggesting that women with BC need a wide range of support. To understand the challenges Japanese women encounter following a diagnosis of BC, we holistically explored decisions women perceived themselves to have made following such a diagnosis.

**Methods:**

This was a cross-sectional, internet-based study comprising open-ended question. Qualitative content analysis was employed on the 1,158 free descriptive responses obtained from 549 participants. The frequencies of decisions were compared according to age at diagnosis using the chi-square test.

**Results:**

Approximately 80% of the participants reported having made some decisions. These decisions were separated into 14 categories: two categories were related to medical care and 12 were related to sociopsychological decisions. The frequency of sociopsychological decisions was higher than that of medical care decisions. About two-thirds of participants reported having made more than two decisions, and about one-third reported having made both medical and sociopsychological decisions. The decisions made by women varied based on age group at diagnosis. The lower the age group at diagnosis, the higher was the frequency of decisions related to both medical care and sociopsychological matters. Participants who were diagnosed with BC at a younger age were more likely to encounter a greater number of sociopsychological decisions, such as those concerning employment, fatality, and marriage, compared with those who were diagnosed at an older age.

**Conclusions:**

This analysis of open-ended questions suggests that Japanese women diagnosed with BC have a wide range of support needs that vary according to their age group at diagnosis.

## Introduction

Living with breast cancer (BC) involves making many decisions, which immediately follow the diagnosis of BC. These decisions concern not only medical matters, but also psychosocial ones, suggesting that women with BC need a wide range of support. Previous research investigating the impact of BC on Japanese women has focused on different aspects of a patient’s life. For instance, almost 70% of adolescent and young adult BC survivors have been found to face issues related to reproductive function, with some giving up their hope of bearing a child despite wishing to have children (Furui et al., 2019). The positive and negative effects of informing their school-age children of their illness have also been articulated for both the women and their children, (Yoshida et al., 2010). About one-third of women tend to resign from their jobs on being diagnosed with BC (Saito et al., 2014). Moreover, the number of women with BC searching for information on BC on Japan’s largest cancer information website in order to make medical decisions has been found to be higher than that of patients with any other type of cancer, and 22% of these women have searched for information about metastasis/recurrence (Okuhara et al., 2018). BC has been seen to affect body image and sexual function among the Japanese, especially among those who have undergone a total mastectomy (Adachi et al., 2007). Collectively, these researches suggest that decision-making or problem-solving related to BC diagnosis or BC treatment is a long and stressful process. Hence, it is likely that women diagnosed with BC are compelled to make more than one decision simultaneously. However, little attention has been paid to the decisions Japanese women have to make under these circumstances, or to the entirety of their experience after being diagnosed with BC. 

Accordingly, the purpose of this study was to holistically explore the decisions made by Japanese women. The findings of this study are expected to foster a better understanding of the challenges encountered by Japanese women when they are diagnosed with BC.

## Materials and Methods

*Respondents and data collection*

We conducted an anonymous, cross-sectional internet survey with a convenience sample of Japanese female BC survivors who were registered with an online marketing research company (Rakuten Insight Inc. Osaka, Japan). The eligibility criteria were: (1) being diagnosed with BC, (2) having had primary treatment for BC including surgery in Japan, and (3) being older than 20 years of age at the time of taking the survey. Women who self-reported a history of BC to the company were invited to participate in this survey by email between February 15 and 18, 2019. 

A total of 550 women completed the online survey. We excluded one questionnaire as it contained an incomplete sentence in the open-ended question section. Thus, we analyzed 1,158 free descriptive responses from 549 participants.

*Measurements*

The self-administered survey consisted of multiple-choice and open-ended questions. The multiple-choice questions sought to obtain information on the sociodemographic characteristics of age, marital status, household income, employment status, youngest child at diagnosis and education; and the cancer-related characteristics of BC stage, type of BC treatment including surgery, and presence/absence of BC recurrence and metastasis. The open-ended question asked from the participants was: “From the time you were diagnosed with breast cancer to the present, please list at the most three things that you have decided because you got sick and that have remained in your mind, regardless of whether or not they were directly related to treatment.” 

*Qualitative content analysis*

All responses were grouped by content based on the co-occurrence of frequently appearing words using KH Coder version 3 (Higuchi, 2016; Higuchi, 2017). After this computerized coding, we categorized the groups containing the responses “nothing in particular” and “I do not remember” as “no decision.” Further, we named the group containing the verbs “regret,” “fear,” and “worry” as “thought,” and separated it from the “decision” category. Nouns reflecting a situation beyond the patient’s control, such as “recurrences,” “metastasis,” or “prognoses” were categorized into “mindset” if these words were concomitant with “changed lifestyle” or “be careful.” Two of the researchers (KY and M Nakashima) discussed all coding process and finally identified 14 categories and nine subcategories of decisions made by Japanese women after being diagnosed with BC.

*Statistical analysis*

The chi-square and Fisher’s exact test were used for analysis. Majorly, two comparison were made: the differences in the characteristics of the participants were compared by the presence or absence of decisions/thoughts, snd the frequencies of decisions in each category were compared by age group at diagnosis. Statistical significance was set at p < .05. The analyses were conducted using SPSS version 24 (IBM Corporation, Armonk, NY, USA).

## Results

*Characteristics of the participants*

The sociodemographic and cancer-related characteristics of the participants are shown in Table 1 by the presence or absence of decisions/thoughts. Of the 549 study participants, 78 (14.0%) did not report any decision or thought since being diagnosed with BC. Compared to their counterparts, participants who were highly educated or had higher household income at diagnosis were more likely to recognize that they had made some decisions or had thoughts since being diagnosed with BC. Women who did not remember their cancer stage at diagnosis and women who received radiation therapy were more likely to have not made any decisions.

*Content of the decisions*

We extracted two core categories of decisions made by Japanese women after being diagnosed with BC (Table 2 and the detail is found in the Supplementary Table 1): those regarding medical care and those regarding sociopsychological matters. The core category of medical care was subdivided into the following two categories: “BC treatment,” and “Medical care other than treatment.” Further, while “BC treatment” contained the subcategories of “Surgery” and “Chemotherapy,” “Medical care other than treatment” contained the subcategories of “Selection of a hospital or physician,” “Obtaining a second opinion,” and “Trusting doctor.” About half of the participants (47.0%) reported making decisions in the medical care category. Selection of the surgical procedure, including whether to undergo reconstructive surgery, was the most frequently reported decision concerning BC treatment (23.3%), followed by decisions regarding chemotherapy (8.7%). Participants faced decisions not only on whether to receive chemotherapy but also on whether to continue or terminate chemotherapy during treatment. 

The core category of sociopsychological matters comprised the following 12 categories: “Mindset,” “Family matters,” “Employment,” “Financial matters,” “Lifestyle modification,” “Informing others about BC,” “Pregnancy/childbirth,” “Preparing for one’s death”, “Appearance,” “Obtaining information,” “Marriage/divorce,” and “Seeking help.” Nearly two-third of participants (65.6%) made some sociopsychological decisions. The most frequent (27.5%) sociopsychological decision related to determining a new mindset, such as one’s attitude toward life with BC and BC treatment, hoping for positivity in life, and planning for the future. In family matters (24.4%), about half of the decisions reported were related to children. Decisions regarding employment concerned both the continuation and discontinuation of employment (23.7%). Decisions regarding financial matters concerned both medical and living expenses (14.4%). Some participants decided to change their lifestyle, such as by improving their eating and exercise habits, giving up smoking, incorporating self-care, or living environmentally (7.7%). Participants reported making decisions relating to whom they should inform about their disease (including family members), and when they should provide this information (5.8%). Younger survivors often faced decisions regarding pregnancy/childbirth, such as whether to opt for fertility preservation or infertility treatment, or whether to have a child in the future (2.4%). Some women made decisions regarding their attitudes toward their death and putting their house in order (3.5%). Decisions regarding appearance included not only working on looking as they did before surgery, but also on dressing up more than they did before their diagnosis (2.2%). Nine participants decided to obtain information regarding BC and BC treatment or information on making their will and tackling the question of inheritance (1.6%). Seven participants decided to start or end a marriage (1.3%). Five participants decided to ask someone for help in overcoming the difficulties caused by BC and BC treatment, or for encouragement in continuing treatment for BC (0.9%). A few women also reported thoughts of fear, worry, and regret following a BC diagnosis (Supplementary Table 2). 

Table 3 presents the frequencies of the core categories of the decisions by number of responses. Of the 549 study participants, 443 (80.7%) reported making some decisions, 348 (63.4%) reported making more than two decisions, and 175 (31.9%) reported making both medical and sociopsychological decisions since being diagnosed with BC. 

*Influence of age on decisions*

Table 2 shows the influence of age at diagnosis on the content of decisions. Overall, lower the age group at diagnosis, the higher was the frequency of decisions relating to both medical care and sociopsychological matters. Statistical differences between age groups were observed in sociopsychological decisions overall, and specifically in decisions regarding employment, pregnancy/childbirth, and marriage/divorce. Participants who were diagnosed with BC at a younger age were more likely to encounter a greater number of sociopsychological decisions, such as those concerning employment, fatality, and marriage, compared with those who were diagnosed at an older age. A similar trend was observed in the frequency of decisions regarding the selection of BC treatment and financial matters. In contrast, the higher the age group at diagnosis, the higher was the frequency of decisions regarding medical care other than surgery.

**Table 1:** Characteristics of Participants by Presence or Absence of Decisions/Thoughts (n=549)

	Total	Decisions/ Thoughts (n, %)		p-value
	(n, %)	None	Some	
Age at diagnosis				
≤ 39	101 (18.4)	11 (14.1)	90 (19.1)	0.522
40–49	257 (46.8)	40 (51.3)	217 (46.1)	
≥ 50	191 (34.8)	27 (34.6)	164 (34.8)	
Marital status at diagnosis				
Single	133 (24.2)	19 (24.4)	114 (24.2)	0.964
Married	391 (71.2)	55 (70.5)	336 (71.3)	
Other	25 (4.6)	4 (5.1)	21 (4.5)	
Education				
≤ High school	169 (30.8)	33 (42.3)	136 (28.9)	0.017
> High school	380 (69.2)	45 (57.7)	335 (71.1)	
Household income at diagnosis				
< ¥5,000.000	276 (50.3)	50 (64.1)	226 (48.0)	0.008
≥ ¥5,000.000	273 (49.7)	28 (35.9)	245 (52.0)	
Employment status at diagnosis				
Regular employment	138 (25.1)	11 (14.1)	127 (27.0)	0.093
Non-regular employment	179 (32.6)	30 (38.5)	149 (31.6)	
Housewife	165 (30.1)	28 (35.9)	137 (29.1)	
Other	67 (12.2)	9 (11.5)	58 (12.3)	
Child at diagnosis				
No child	215 (39.2)	40 (51.3)	175 (37.2)	0.113
With a child (or children)	334 (60.8)	38 (48.7)	296 (62.8)	
Family history of breast cancer				
No	410 (74.7)	61 (78.2)	349 (74.1)	0.440
Yes	139 (25.3)	17 (21.8)	122 (25.9)	
BC stage				
0	81 (14.8)	7 (9.0)	74 (15.7)	0.003
I	195 (35.5)	31 (39.7)	164 (34.8)	
II	139 (25.3)	14 (17.9)	125 (26.5)	
≥ III	62 (11.3)	6 (7.7)	56 (11.9)	
Other (do not remember, etc.)	72 (13.1)	20 (25.6)	52 (11.0)	
Surgery				
Total Mastectomy	230 (41.9)	28 (35.9)	202 (42.9)	0.121
Breast Conserving Surgery	277 (50.5)	47 (60.3)	230 (48.8)	
Other	42 (7.7)	3 (3.8)	39 (8.3)	
Recurrence/metastasis of BC				
No	471 (85.8)	70 (89.7)	401 (85.1)	0.281
Yes	78 (14.2)	8 (10.3)	70 (14.9)	
Radiation therapy				
No	233 (42.4)	24 (30.8)	209 (44.4)	0.016
Yes	316 (57.6)	54 (69.2)	262 (55.6)	
Chemotherapy				
No	319 (58.1)	51 (65.4)	268 (56.9)	0.160
Yes	230 (41.9)	27 (34.6)	203 (43.1)	
Hormone therapy				
No	157 (28.6)	21 (26.9)	136 (28.9)	0.724
Yes	392 (71.4)	57 (73.1)	335 (71.1)	

**Table 2:** Decisions and Their Frequencies Made by Women with BC Following Diagnosis and by Age Group at Diagnosis (n=549)

	Total	Age groups at diagnosis (n, %)			p-value
Categories of decisions	(n, %)	≤ 39	40–49	≥ 50	
I. Decisions regarding medical care	258 (47.0)	51 (50.5)	122 (47.5)	85 (44.5)	0.608
1. BC treatments	209 (38.1)	45 (44.6)	99 (38.5)	65 (34.0)	0.208
2. Medical care other than treatments	90 (16.4)	12 (11.9)	43 (16.7)	35 (18.3)	0.360
II. Decisions regarding sociopsychological matters	360 (65.6)	78 (77.2)	168 (65.4)	114 (59.7)	0.011
3. Mindset	151 (27.5)	25 (24.8)	57 (22.2)	46 (24.1)	0.833
4. Family matters	134 (24.4)	32 (31.7)	58 (22.6)	44 (23.0)	0.168
5. Employment	130 (23.7)	34 (33.7)	60 (23.3)	36 (18.8)	0.018
6. Financial matters	79 (14.4)	19 (18.8)	38 (14.8)	22 (11.5)	0.233
7. Lifestyle modification	42 (7.7)	12 (11.9)	15 (5.8)	15 (7.9)	0.152
8. Informing others about BC	32 (5.8)	6 (5.9)	12 (4.7)	14 (7.3)	0.493
9. Pregnancy/childbirth	13 (2.4)	8 (7.9)	5 (1.9)	0 (0.0)	<0.001
10. Preparing for one’s death	19 (3.5)	2 (2.0)	9 (3.5)	8 (4.2)	0.617
11. Appearance	12 (2.2)	3 (3.0)	8 (3.1)	1 (0.5)	0.150
12. Obtaining information	9 (1.6)	0 (0.0)	5 (1.9)	4 (2.1)	0.354
13. Marriage/divorce	7 (1.3)	4 (4.0)	3 (1.2)	0 (0.0)	0.016
14. Seeking help	5 (0.9)	1 (1.0)	3 (1.2)	0 (0.0)	0.524

**Table 3:** Categories of Responses and Their Frequencies (n=549)

	Number of decisions made (frequency of responses)		
	1 (n=118)	2 (n=100)	3 (n=253)
Categories of responses (top three)	Decisions regarding sociopsychological matters (n=57, 48.3%)	Decisions regarding medical care + Decisions regarding sociopsychological matters (n=44, 44.0%)	Decisions regarding medical care + Decisions regarding sociopsychological matters (n=123, 48.6%)
	Decisions regarding medical care (n=38, 32.2%)	Decisions regarding sociopsychological matters (n=32, 32.0%)	Decisions regarding sociopsychological matters (n=70, 27.7%)
	Thoughts following BC diagnosis (n=20, 16.9%)	Decisions regarding medical care (n=11, 11.0%)	Decisions regarding medical care (n=23, 9.1%)

## Discussion

With the purpose of comprehensively understanding the challenges Japanese women encounter following a diagnosis of BC, we explored the responses of Japanese women with BC to an open-ended questionnaire about decisions they made since being diagnosed with BC. More than 80% of the participants reported that they had made some decisions. Qualitative coding methods identified 14 categories of decisions: two related to medical care and 12 related to sociopsychological decisions. About one-third of participants reported making both medical and sociopsychological decisions. Women’s decisions varied according to their age group at diagnosis. Younger women were more likely to make a greater number of sociopsychological decisions, especially decisions concerning employment, financial matters, fertility, and marriage.

Decision-making that is not directly related to BC treatment may be significant for patients. The current study shows that more than 80% of participants reported making some decisions since being diagnosed with BC; 47% decided about medical care, of which 38% reported making decisions about BC treatment. Between 70% and 80% of Japanese BC patients have been found to decide BC treatment alone or collaboratively with doctors (Nakashima et al., 2012; Shimizu et al., 2019; Yamauchi et al., 2019). In this regard, considering that a patient playing an active or collaborative role in decision-making for treatment has made the decision themselves, fewer women reported decision-making regarding treatment in our study compared with the results reported in previous studies. The open-ended question we used in this study may have led to this difference between the results of the current and previous studies. We asked the study participants to list the decisions that have remained in their minds, suggesting that selecting BC treatment may not have been a significant event for the patients, or that they may have faced more difficult decisions than those regarding BC treatment. However, 66% of participants in this study reported making decisions regarding sociopsychological matters, suggesting that the influence of sociopsychological decisions is felt long after the completion of primary treatment. The other possibility is that some participants may not have recognized that they had participated in choosing their BC treatments, as approximately 26–43% of these decisions have been found to be conducted collaboratively with doctors (Nakashima et al., 2012; Shimizu et al., 2019; Yamauchi et al., 2019). There is a positive association between involvement in treatment decision-making and quality of life (QOL) in cancer patients (Hack et al., 2006; Andersen et al., 2009; Atherton et al., 2013; Andersen et al., 2018), suggesting that the manner in which these psychosocial decisions are made, along with the decisions themselves, contribute to increasing a patients’ QOL. Even though sociopsychological status after primary care significantly influences cancer patients’ QOL (Kobayashi et al., 2008; Konieczny et al., 2020), to the best of our knowledge, no research has been conducted on how patients’ involvement in sociopsychological decision-making or how the type of sociopsychological made by them affect their QOL afterward. Further studies are needed to investigate how sociopsychological decisions impact patients and their lives longitudinally.

Open-ended questions such as those used in the current study could elicit more holistic responses on what women do and need after being diagnosed with BC, especially with regard to sociopsychological decisions. In this study, decisions related to employment, finance, and family matters were the frequently reported sociopsychological decisions, while decisions related to fertility and marriage were characteristic of younger participants. These items have been articulated as sociopsychological challenges, and factors associated with them have been investigated in order to develop better assistance in the lives of Japanese BC survivors (Yamaguchi et al., 2007; Takahashi et al., 2008; Yoshida et al., 2010; Saito et al., 2014; Umezawa et al., 2015; Hisamura et al., 2018; Takahashi et al., 2018; Takeuchi et al., 2018; Furui et al., 2019). Additionally, our study found that determining a new mindset accounted as a frequently made decision across all age groups. In this regard, planning for the future is one of the biggest problems experienced by Japanese cancer survivors (Hisamura et al., 2018; Takeuchi et al., 2018). Preparing mentally for life during and after BC treatment may make life easier for BC survivors. This requires an investigation into determining patients’ mindset, despite the variable being less emphasized among cancer-related difficulties.

Patients’ needs may differ by situation and may be wide-ranging. Our study reveals that about two-third of participants made more than two decisions on matters both related and unrelated to medical care. Yamaguchi et al. extracted 16 categories of decisions, including matters both related and unrelated to medical care, based on the distress and inquiries of 48,031 pooled cases of Japanese cancer patients from a questionnaire survey and consultation service (Yamaguchi et al., 2007). In their study, the frequency of decisions regarding sociopsychological matters differed considerably depending on the method of data collection. The results from their questionnaire survey corroborate with the results of our study: the frequency of decisions regarding sociopsychological matters was higher than the frequency of decisions regarding medical care in both cases; however, the results from the consultation service at the hospital showed the opposite trend. This suggests that patients’ needs differ between clinical and non-clinical settings. Furthermore, many participants in our study reported making more than two decisions about medical care or medical care combined with sociopsychological matters. This suggests that collaboration between professionals’ support and non-medical support, such as that offered by peers and workplaces, is needed to comprehensively help women with BC (Umezawa et al., 2015; Takahashi et al., 2018). 

The support needed for BC patients depends on their age at diagnosis. Our study revealed that the lower the age group at diagnosis, the higher was the frequency of sociopsychological decisions. Age differences were observed in the categories of sociopsychological decisions: younger participants decided how to take care of, and whom to ask for help while taking care of children during hospitalization and treatment, while older participants were more likely to do so for a husband or elderly parents. Decisions relating pregnancy/childbirth and marriage/divorce were made only by participants who were younger than 49 years of age. Patients in this age group considered the influence on pregnancy/childbirth and their sex life when they chose their cancer treatment (Yamauchi et al., 2019). This shows that measures for providing support that match the age or life stage of the patients need to be developed. 

This study had several limitations. First, our findings are likely to be limited in generalizability because of selection bias due to the nature of internet-based, self-administered surveys. Second, 14% of the participants did not recall or report making any decisions. This may be because of the length of time that had passed since they were diagnosed with BC, suggesting a recall bias, and the rare practice of autonomous decision-making amongst Japanese at the time of their diagnosis. Third, even though we asked participants what kind of decisions they made, 12% of them expressed their thoughts of fear, worry, and regret. The questions might remind them, more strongly than they had anticipated, of their feelings when they made some decision or thoughts brought about by the consequence of those decisions. As such, these responses provided by them might be linked to the decisions made by them, but we separated these responses from the decision category. Hence, we might have missed extracting some decisions from the participants’ responses.

Despite these limitations, this study highlights that when Japanese women are diagnosed with BC, they make a wide range of decisions that include both medical care and sociopsychological matters. These decisions vary according to their age at the time of diagnosis. Comprehensively looking at the needs of women diagnosed with BC will provide information to physicians and other medical specialists about medical and social support. This information will also help women who are newly diagnosed with BC in preparing for the challenges they might encounter.

## Author Contribution Statement

KY participated in the study design, coordination of data collection, analysis and interpretation of the data, and drafting the manuscript. M Nakashima participated in the study design, data analysis and interpretation of the data. N Nakao participated in drafting the manuscript and proofreading the final submission. All authors reviewed the results and approved the final version of the manuscript.
